# Domestic violence among healthcare professionals in Sweden - Prevalence, associations with asking older patients about abuse and reactions to participating in training on abuse of older people

**DOI:** 10.1186/s12913-026-15440-y

**Published:** 2026-08-22

**Authors:** Johanna Simmons, Mikael Ludvigsson

**Affiliations:** 1 Department of Health, Medicine, and Caring Sciences, Linköping University, Linköping, Sweden; 2 Clinical Department of Geriatrics and Palliative Medicine in Linköping, Region Östergötland, Linköping, Sweden; 3 Department of Biomedical and Clinical Sciences, Linköping University, Linköping, Sweden; 4 Clinical Department of Psychiatry in Linköping, Region Östergötland, Linköping, Sweden

**Keywords:** Intimate partner violence, Family violence, Elder abuse, Abuse of older people, Medical education, Re-traumatisation, Trauma informed care

## Abstract

**Background:**

Different forms of domestic violence, including abuse of older people (AOP), are prevalent and associated with poor health outcomes. The healthcare system is therefore often considered an important context for identifying victims of violence and providing referrals. However, healthcare professionals may also have their own experiences of domestic violence. The aims of this study were to: (1) investigate the prevalence of domestic violence, specifically intimate partner violence (IPV) and violence by other family members, among healthcare professionals in Sweden; (2) examine whether such exposure was associated with asking older patients about abuse, and (3) explore whether (a) experiences of domestic violence were associated with negative feelings when participating in AOP training and, (b) whether such negative feelings were associated with perceiving the training as valuable.

**Methods:**

Cross-sectional survey data were collected from healthcare professionals who participated in the Responding to Elder Abuse in GERiAtric care educational intervention trial (REAGERA Edu).

**Results:**

IPV was reported by 21.2% of participants (women 23.0%, men 8.8%), while 20.7% reported experiences of violence by other family members (women 21.6%, men 14.7%). Reporting multiple victimisation, i.e., both IPV and family violence, was associated with increased odds of asking patients about AOP (OR 2.45; 95% CI 1.14–5.28). Further, experiences of IPV only (OR = 2.98; 95% CI 1.54–5.77) and multiple victimisation (OR = 3.43; 95% CI 1.43–8.21) was associated with reporting negative feelings when participating in AOP training, but most respondents who reported negative feelings also recognised the value of the training.

**Conclusions:**

Experiences of domestic violence are common among healthcare professionals and are associated with a greater likelihood of asking patients questions about AOP and with negative feelings when participating in AOP training. A trauma-informed approach is important in training professionals to improve readiness to care for victims of AOP in healthcare while simultaneously avoiding re-traumatising healthcare professionals.

**Trial registration:**

ClinicalTrials.gov Identifier NCT05065281. Retrospectively registered on September 28, 2021. The trial registration concerns the primary outcomes of the REAGERA educational intervention, i.e., effects of the intervention, while the current study report on secondary aims not mentioned in study protocol.

**Supplementary Information:**

The online version contains supplementary material available at 10.1186/s12913-026-15440-y.

## Background

Different forms of domestic violence, including intimate partner violence (IPV) and abuse of older people (AOP), are considered a global public health problem due to their high prevalence and long-lasting negative health effects [13, 36, 42, 45]. Depending on the methodology used, the exact prevalence of different forms of violence varies considerably between studies; however, exposure to violence is generally considered a common experience over the life course [15, 29]. For example, in Swedish studies including respondents between 16 and 85 years, the lifetime prevalence of emotional IPV has been reported between 11% and 41% for women and between 4% and 37% for men [29]. Studies indicate that experiences of violence among healthcare professionals are at least as common as in the general population, and possibly even more common [20, 22]. A global meta-analysis reported the pooled lifetime prevalence of IPV among healthcare professionals in high income countries to be 20.7% [10]. The prevalence of IPV among healthcare professionals in Sweden has been reported in two studies. One study, restricted to female healthcare professionals at one hospital who had been exposed to violence by male partners, reported a lifetime prevalence rate of 23.5% [35]. The second study, restricted to primary care nurses, used only one item about exposure to IPV and reported a lifetime prevalence of 13% [37]. Neither study investigated exposure to other forms of family violence beyond IPV.

In this study, we use the term “IPV” when referring to any form of violence by a current or former partner, “family violence” when referring to violence by family members other than intimate partners, and “domestic violence” when referring to IPV or family violence collectively. The terms “abuse” and “violence” are used interchangeably. It is important to consider experiences of domestic violence among healthcare professionals for a variety of reasons. Exposure to IPV has been reported to affect work performance. For example, perpetrators are known to directly interfere in the workplace through harassment and stalking behaviours [2, 10, 11]. Also, domestic violence may lead to both mental and physical ill-health, including depression and post-traumatic stress disorder (PTSD) which in turn may lead to sickness absence [12, 17, 36, 42]. Facing patients with experiences of violence has also been reported to trigger negative reactions and re-traumatisation for some healthcare professionals with personal experiences of violence [11]. Studies also indicate that personal experiences of violence may shape the response of healthcare professionals when treating patients exposed to IPV [11]. However, findings across different studies have varied, with some reporting an improved ability to identify patients subjected to violence [11], while others report no difference [26]. It is therefore important to understand if, and how, personal exposure to domestic violence among healthcare professionals affects the identification of patients exposed to violence, and whether this affects the care they provide, as the care system is often considered an important context for identifying victims of violence. For example, in Sweden, the National Board of Health and Welfare has issued a statement that all patients showing sign or symptoms of domestic violence should be asked questions about the underlying causes of these symptoms (Swedish National Board on Health and Welfare [38] .

It is possible that the association between healthcare professionals’ own exposure to violence and their ability to identify patients exposed to violence varies between different forms of violence. For example, professionals with personal experiences of childhood abuse may be better equipped to recognise signs of childhood abuse, but not necessarily AOP. The term AOP refers to a single or repeated act, or lack of appropriate action, that causes harm or distress to an older adult and occurs within a relationship with an expectation of trust (WHO [43]), . In contrast to domestic violence, AOP includes neglect as a central form of abuse and encompasses perpetrators beyond family members, including health and social care staff. A global review and meta-analysis found that one in six community-dwelling older adults reported some form of abuse in the last 12 months [44]. In Sweden, past-year prevalence estimates have ranged from 3% to 30%, with much of this variations likely reflecting methodological differences in measurement and sampling strategies [1, 18, 25]. However, many victims are reluctant to seek help and may do so only when the abuse is perceived as unbearable or when they fear for their safety [14]. Healthcare professionals play a crucial role in identifying all forms of violence, but this is especially important for older patients, for whom care dependence may be a barrier to seeking help [9]. To the best of our knowledge, the associations between healthcare professionals’ personal history of violence and patterns of identifying AOP has not been previously investigated.

It is often suggested that education and training about violence is needed to improve healthcare professionals’ response to victims of violence, including AOP [33, 39]. Still, many professionals lack training on IPV in general and AOP specifically. In a previous study, we found that only 27% of staff working in an internal medicine and geriatric department in Sweden had received any training on AOP. However, having training on both IPV and AOP was strongly associated with asking older patients about exposure to violence [23]. Since 2018, training on the handling of domestic violence cases has been included as part of the qualitative targets in healthcare education programmes in Sweden (e.g., in the medical, nursing, and physiotherapist training curricula). However, while training on violence is needed to improve care, it also has the potential to trigger negative reactions among participants, especially those with a personal history of exposure to violence. For example, one study that included students in a course on trauma treatment found that the training led to re-experiencing own trauma, intrusive thoughts, avoidance behaviours, and hyperarousal among trauma survivors [28]. The potential negative effects of training on care for victims of violence have primarily been discussed in relation to students [5, 6, 24, 28]. However, there are also reports that educating healthcare professionals about different forms of violence can trigger negative reactions among those with personal experiences of violence. Some participants may even realise for the first time during the training that they themselves are victims of IPV [11]. Though training on violence is important for healthcare professionals, it is also essential to avoid re-traumatisation when conducting training. It is therefore important to gain a better understanding of how healthcare professionals’ own experiences of violence affect the care they provide to patients with experiences of violence, as well as potential negative effects of training on violence. Some studies have begun to explore these associations for IPV, but they remain unexplored in relation to AOP. Therefore, the aims of this study were to:

1. Investigate the prevalence of personal exposure to IPV and violence by other family members among healthcare professionals in Sweden.
2. Investigate whether healthcare professionals’ own experiences of domestic violence were associated with asking older patients questions about AOP.
3. Investigate (a) whether healthcare professionals’ own experiences of domestic violence were associated with reporting negative feelings when participating in training on AOP and (b) whether reporting negative feelings of participation were associated with perceiving the training as valuable.

## Materials and methods

### Participants and procedures

This study is part of the Responding to Elder Abuse in GERatric care educational intervention (REAGERA edu), which examines the effects of AOP training on healthcare professionals [19, 33]. The intervention in the study consisted of a full day of AOP training that combined lectures, videos, groups discussions, forum theatre and reflections about how to improve clinical practice in relation to AOP. Forum theatre [7] a participatory form of theatre, was conducted together with three professional actors. First, the actors enacted a challenging healthcare encounter, involving AOP that did not end well. Participants were then invited to intervene by replacing the healthcare profession in the scene or suggesting alternate responses. Together the actors and participants explored different ways of managing a difficult situation, often illustrating that situations initially perceived as almost impossible to manage may be approached in multiple ways. All parts of the training day included elements about older adults exposure to both domestic violence and abuse by staff. In the forum theatre, three scenes were enacted, each involving a different perpetrator: an adult child, an intimate partner and a staff member. The training thus relied heavily on interactive methods, intended to evoke emotions and engagement with the issue and to achieve embodied knowledge. However, this may also have elicited flashbacks and negative recollections among participants with personal experiences of violence, which is explored in this study. To minimise potential negative consequences, eligible participants received information in advance about the training content, planned learning activities and the study. At the start of the training day, it was acknowledged that the training could elicit negative recollections and participants were encouraged to be attentive to each other’s emotional reactions during the day. They were also invited to speak with the educators if needed, one of whom is a consultant psychiatrist. In addition, contact information was provided for a regional crisis therapist not involved in the research project, as well as for societal support organisations for victims of violence.

**Fig. 1:**
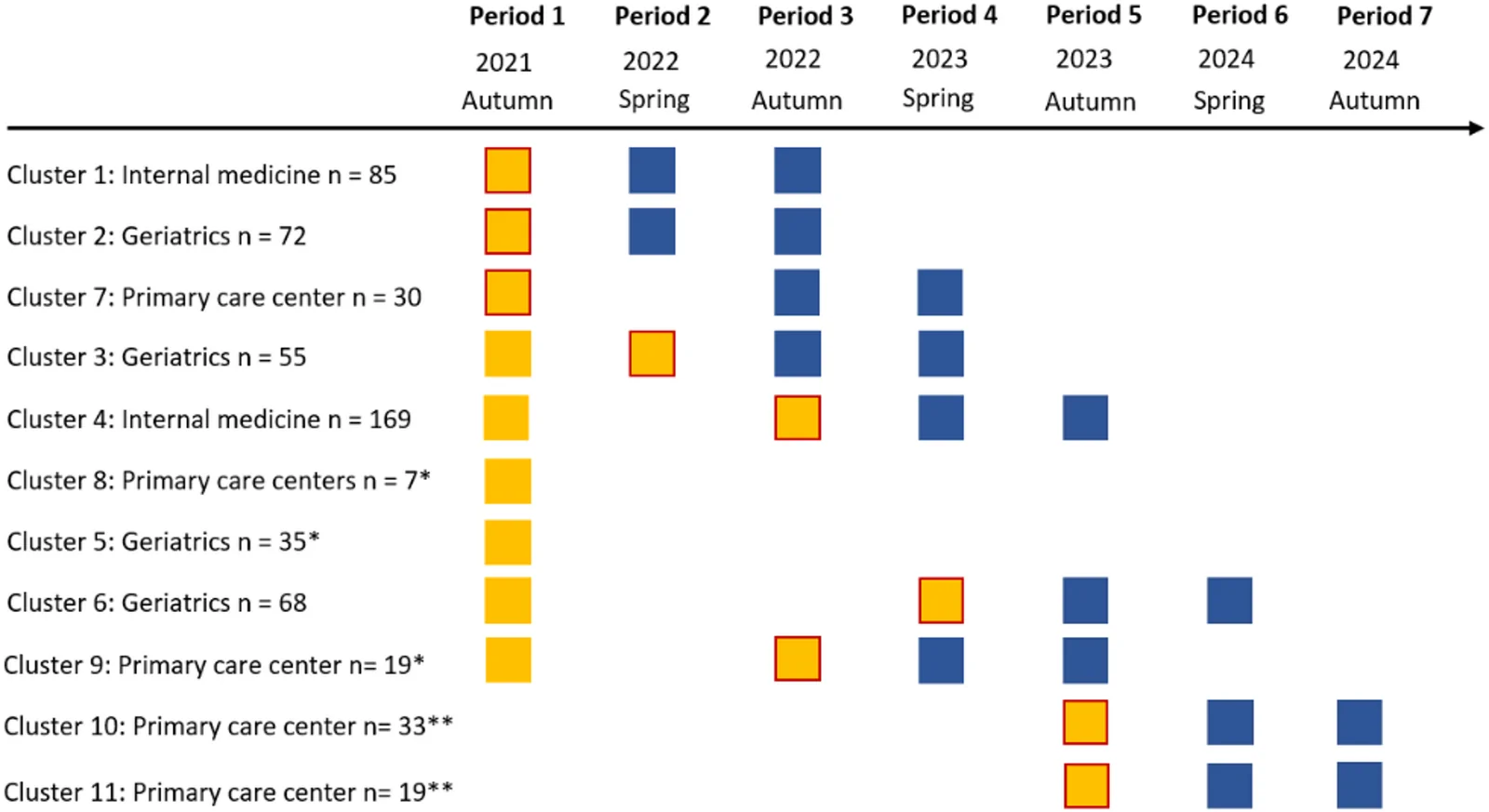
Design of the study and data collection points. An incomplete stepped wedge trial was conducted including data collection pre-intervention and post-intervention. Number of respondents given in the figure represents number of clinically active staff in each cluster answering at least one pre-intervention measure (*n* = 592). Yellow squares with red contour = educational day with both pre-intervention and post-intervention measure; yellow squares without red contour = pre-intervention measure at baseline; blue squares= follow-up measure at 6–8 months and 12–15 months. The numbering of clusters in the figure is in accordance with the study protocol. As the implementation took place in another order than planned, the numbering of clusters is thus not in ascending order in the figure. *Clusters that cancelled their participation between baseline measure and planned intervention. In cluster 9, six staff participated in the intervention together with cluster 4. ** Clusters that replaced clusters 8 and 9. Cluster 11 constitutes a mix of participants from various primary healthcare centres

The content of the training, as well as the design of the study, including the sample size calculation, is described in detail in the study protocol [19]. In brief; the REAGERA-Edu was designed as a stepped wedge trial [8], in which the intervention (training day) was rolled out sequentially to clusters of participants between autumn 2021 and autumn 2023. An overview of the study design can be found in Fig. 1. Originally, the clusters consisted of primary healthcare centres (*n* = 3) and in-hospital geriatric (*n* = 4) or internal medicine (*n* = 2) care units in southeast Sweden. The intervention was planned as part of the continuing education programme for the clinics. As far as possible, all staff members were scheduled to participate in the training in relation to their clinical responsibilities. However, one geriatric clinic and two of the original primary healthcare centres (clusters 5, 8, 9, Fig. 1) withdrew between the baseline measure and the intervention, citing organisational changes and staff shortages. The geriatric clinic withdrew late and could therefore not be replaced. The primary healthcare centres were replaced by (a) one new primary healthcare centre (cluster 10, Fig. 1) and (b) an open invitation to staff at all primary healthcare centres in one region to participate in a designated training day (cluster 11, Fig. 1). In addition, six professionals working at one of the primary healthcare centres that withdrew (cluster 9) participated in training days conducted with one of the internal medicine clinics.

The inclusion criterion for the REAGERA-Edu was participation in the training day. For this study, those who answered the baseline measure but did not participate in the intervention were also included. All staff working at the participating clinics were eligible, including doctors, nurses, assistant nurses, and other professionals such as physiotherapists, dietitians, occupational therapists, counsellors, and psychologists. Though all staff at the participating clinics were invited to participate in the study, acceptance was not a prerequisite for participating in the training. Informed consent was obtained by responding to the first item in the online survey used for data collection, which was mandatory. The exclusion criterion was not working with older patients in a clinical capacity. Hence, at some clinics, administrative staff and managers attended the training day but were not included in the study, since they did not work with older patients in a clinical capacity.

Data were collected on two occasions prior to the intervention: at baseline, in autumn 2021, and on the morning of the training, before it commenced. The timing of the latter varied between clusters, from autumn 2021 to autumn 2023 (Fig. 1). Post-intervention data were collected at three time points: the afternoon after completing the training, six months post-intervention, and twelve months post-intervention (Fig. 1). For this study, pre-intervention data were combined to form a cross-sectional dataset. The first response given by a respondent was used, meaning that when a respondent answered both at baseline and on the morning of the training, baseline data were used. This approach was chosen because items on background characteristics, including own exposure to violence, were assessed only once. To ensure consistency in the cross-sectional analysis, we therefore used each participant’s first response, when all relevant items had been completed. To investigate aim 3, concerning negative feelings elicited by the training, we also used data from the first post-intervention measure, which was collected on the afternoon of the training session. Data from the 6- and 12-month follow up measures were not considered in the current study.

### Measurement

The REAGERA-P questionnaire (Responding to Elder Abuse in Geriatric Care – Provider questionnaire) was used for data collection [32]. It has previously been validated among healthcare professionals in Sweden and found to be a reliable measure of professionals’ preparedness to care for older adults subjected to abuse. For the REAGERA-Edu trial, the questionnaire was extended to include new items. In the first data collection, these concerned personal experiences of violence, while in the first post-intervention measurement, they concerned experiences of participating in the training. All new items were validated using cognitive interview techniques [4]. In the cognitive interviews, participants were asked to read the items in the questionnaire and describe their thought process aloud. The method was used to capture the cognitive process when reading and answering the items, thereby assuring comprehensibility.

The following items were used to capture personal experiences of different forms of violence:

- Physical violence: “Have you yourself, as a child or as an adult, been subjected to any kind of physical violence? For example, being beaten, kicked, forcibly held or subjected to other physical violence that you perceived as frightening.”
- Sexual violence: “Have you yourself, as a child or as an adult, been subjected to any kind of sexual violence? For example, somebody touching your body against your will or forcing you to perform sexual acts.”
- Emotional violence: “Have you yourself, as a child or as an adult, been subjected to any kind of emotional violence? For example, that somebody repeatedly degraded you, humiliated you or tried to limit your contact with others or decide what you may and may not do.”
- Financial violence: “Have you yourself, as a child or at an adult age, been subjected to any kind of financial or material violence? For example, that somebody exploited you financially or took control of your finances.”

Possible responses to the items about personal experiences of violence were: “No”, “Yes as a child (< 18 years)”, and “Yes as an adult (≥ 18 years)”. Frequency of exposure was not accounted for. If an affirmative answer was given, a follow-up question was asked about the perpetrator with the following possible answers: “A partner or former partner”, “A family member or relative”, “Another person I knew”, and “A completely unknown person”. Only IPV and violence by other family members or relatives were considered in the present study. Responses were coded into several variables based on whether they reported or did not report (1) ever experiencing a specific form of violence (physical, emotional, sexual, or financial) by an intimate partner or other family member, and (2) any form of violence by (a) an intimate partner, (b) a family member or relative, or (c) both an intimate partner and family member/relative. The last category will be referred to as reporting multiple victimisation.

A single question was used to investigate whether participants had experiences of asking older patients about AOP: “Approximately how many times have you asked older patients questions about abuse in the past six months?” Possible response categories were “never”, “one time”, “two times”, “three times”, “four times”, “five times”, “six times”, “seven times”, “eight times”, “nine times”, and “ten or more times”. To enable binary logistic regression analysis and because responses were heavily skewed, responses were dichotomised into: “never” and “at least once”.

To measure whether negative feelings were elicited by the intervention, the following item was used: “To what extent do you feel that different parts of the training day caused negative feelings that you would have preferred to avoid, such as intrusive negative memories, strong feelings of discomfort, or similar experiences?” The question covered four components: the introductory lecture, videos and subsequent discussions, the forum theatre, and the overall experience. Each component had the following response options: “not at all”, “to a small extent”, “to a large extent”, and “to a very large extent”. If a respondent reported “to a large extent” or “to a very large extent” to at least one of the four questions about negative feelings, the participant was classified as having experienced negative feelings. To investigate positive effects of the intervention, a similar four-component item was used: “To what extent do you feel that the different parts of the training day were valuable?” Potential answers were the same as for the item about negative feelings.

In addition, items on sex, occupation, occupational experiences, and previous AOP and domestic violence training were included. To investigate aims 1 and 2, pre-intervention data were used. To investigate aim 3, both pre- and post-intervention data were used.

### Statistical analysis

Statistical analyses were performed using SPSS (version 29; IBM Corporation, USA).

***Aim 1: Prevalence of exposure to violence***: Descriptive analyses were conducted to determine the percentage of healthcare professionals who had experienced the different types of violence. Pearson’s chi square test was used to examine differences between men and women.

As previously mentioned, the intervention was part of the continuing education at most participating clinics, and staff had been scheduled to participate, ensuring that the sample was representative of the clinic. However, this was not the case for some of the primary care participants who participated separately from their clinics (clusters 9 and 11, Fig. 1). Also, some only answered the baseline measure, either because their clinic withdrew from the intervention (clusters 5, 8, and 9, Fig. 1, *n* = 240) or because they were not available for participation during the training day (*n* = 159). Reasons for the latter are unknown, but high staff turnover at the clinics during the two-year intervention period makes it likely that in most cases non-participation was due to discontinuation of employment at the clinic. Together, this was considered a threat to the generalisability of findings concerning prevalence. Therefore, prevalence rates were also calculated separately for (a) respondents participating as part of a clinic, and (b) respondents who either answered only the baseline measure or participated separately from their clinics. Pearson’s chi-square test was used to test for differences in reported prevalence between the two groups.

***Aim 2: Associations between reporting personal exposure to violence and asking older patients about abuse***: A multivariable logistic regression analysis was performed to investigate associations between reporting personal exposure to different forms of violence and asking older patients about AOP. The model was adjusted for background characteristics (sex, occupation, years of occupational experience, and previous training on IPV and AOP). Because some forms of violence were only reported by very few respondents, only the overall variable on the kind of perpetrator was included in the analysis. Respondents’ age and years of occupational experience were highly correlated (Spearman correlation coefficient = 0.662). To avoid multicollinearity, age was excluded in the multivariable analysis since occupational experience was deemed more theoretically relevant.

***Aim 3: Personal experiences of violence and self-reported negative feelings of participating in training.*** A multivariable logistic regression model was conducted with reports of negative feelings as the dependent variable. Background characteristics and experiences of violence were used as explanatory factors. In addition, a cross-tabulation was performed to explore the relationship between reporting negative feelings during the training and perceiving the training as valuable.

### Ethical approval

The study was conducted in accordance with the Declaration of Helsinki concerning research involving human subjects and approved by the Swedish Ethical Review Authority (Registration nos. 2020/02548, 2020/04862, 2021/04179 and 2021/04514). All participants were informed about the research project and provided consent by answering a mandatory question in the REAGERA-P survey regarding participation.

## Results

In total, 659 clinically active healthcare professionals were scheduled to participate in the training days, and 554 (84.1%) participated. The most common reasons for non-participation were sick leave or the need to care for sick children, although some respondents also cited clinical responsibilities. Among participants, 495 answered the survey on the morning of the training day (response rate 89.4%). In addition, the REAGERA-P was sent to 395 staff at baseline who were never scheduled to participate in the training, with 87 responding (response rate 22.0%). Also, five staff who were scheduled to participate in the training but did not attend, and five staff who did attend the training but did not respond to the survey on the morning of the training, answered the baseline measure. Altogether, 592 staff answered the pre-intervention measure (only baseline *n* = 97, only morning *n* = 419, both baseline and morning *n* = 76). The post-intervention survey was answered by 457 of the 554 staff who participated in the training (response rate 82.5%). However, 18 respondents were excluded due to a lack of pre-intervention data, leaving a total sample of 439 respondents post-intervention. Background characteristics of the sample are presented in Table 1.

**Table 1:** Background characteristics of the pre-intervention (*n* = 592) and post-intervention (*n* = 439) samples

Variable	Total sample (*n* = 592)		Answered post-intervention (*n* = 439)	
	*N*	(%)	*N*	(%)
**Sex**				
Female	520	88.0	387	88.2
Male	70	11.8	51	11.6
Other	1	0.2	1	0.2
Missing	1		-	
**Age**				
Up to 34 years old	211	35.6	158	36.0
35–49 years old	196	33.1	145	33.0
50 years or older	185	31.3	136	31.0
Missing	-		-	
**Occupation**				
Assistant nurse	196	33.2	147	33.5
Nurse	227	38.4	184	41.9
Physician	93	15.7	65	14.8
Other*	75	12.7	43	9.8
Missing	1		-	
**Occupational experience**				
Less than one year	48	8.1	41	9.4
1–5 years	139	23.5	101	23.1
5–10 years	108	18.3	80	18.3
More than 10 years	296	50.1	216	49.3
Missing	1		1	
**Previous training AOP**				
No or Don’t remember	496	84.2	364	83.3
Yes	93	15.8	73	16.7
Missing	3		2	
**Previous training DV**				
No or Don’t remember	339	57.6	263	60.2
Yes	250	42.4	174	39.8
Missing	3		2	
**Asked questions about AOP past 6 months**				
Never	469	79.5	346	79.2
1 time	63	10.7	49	11.2
2–3 times	42	7.1	32	7.3
4–6 times	9	1.5	5	1.1
7–9 times	0	-	0	-
10 times or more	7	1.2	5	1.1
Missing	2		2	

Notes: AOP= Abuse of older people, DV= Domestic violence. *Other included e.g. occupational therapists, physiotherapists, dietitians, counsellors and psychologist

### Aim 1: Prevalence of exposure to violence

The reported prevalence of exposure to different forms of violence is presented in Table 2. In total, 35% (*n* = 203) of respondents reported exposure to some form of domestic violence. The sex difference was considerable, with more women (*n* = 190; 37.3%) than men (*n* = 13; 19.1%) reporting any domestic violence exposure (*p* < 0.01). Notably, more women reported experiences of IPV (women *n* = 117, 23.0%; men *n* = 6, 8.8%, *p* < 0.01), while the sex difference regarding violence by other family members (women *n* = 110; 21.6%; men *n* = 10; 14.7%) was not statistically significant (*p* = 0.19). No test was performed for sex differences in relation to specific forms of violence or multiple victimisation due to the small number of men exposed.

**Table 2:** Prevalence of exposure to domestic violence in total sample (*n* = 592) as well as among women (*n* = 520) and men (*n* = 70) separately

	Total sample		Women		Men		*p*-value	
	*N*	%	*n*	%	*n*	%		
**Any domestic violence**								
Yes	203	35.1	190	37.3	13	19.1		< 0.01
No	376	64.9	319	62.7	55	80.9		
**Any IPV**	123	21.2	117	23.0	6	8.8		< 0.01
Physical IPV	50	8.6	49	9.6	1	1.5		
Sexual IPV	37	6.5	36	7.2	1	1.5		
Emotional IPV	96	16.7	91	18.1	5	7.4		
Financial IPV	33	5.8	30	6.0	3	4.4		
**Any family violence**	120	20.7	110	21.6	10	14.7		0.19
Physical family violence	75	13.0	66	13.0	9	13.2		
Sexual family violence	26	4.6	26	5.2	0			
Emotional family violence	59	10.3	52	10.3	7	10.3		
Financial family violence	15	2.6	14	2.8	1	1.5		
**Multiple victimisation**								
No violence	376	64.9	319	62.7	55	80.9		
Only family violence	80	13.8	73	14.3	7	10.3		
Only IPV	83	14.3	80	15.7	3	4.4		
Both family violence and IPV	40	6.9	37	7.3	3	4.4		

Notes: IPV= Intimate partner violence. One respondent did not answer the question about sex and one respondent answered “other”, both were coded as missing on the sex variable. Item non-response for questions about exposure to violence: Physical violence *n* = 13 (2.2%), Sexual violence *n* = 21 (3.5%), Emotional violence *n* = 18 (3.0%), Financial violence *n* = 21 (3.5%)

Reported prevalence rates for respondents who participated as part of a clinic (clusters 1–4, 6–7, and 10; Fig. 1, *n* = 476) and for respondents who either only answered the baseline measure or participated in the training separately from their clinic (clusters 5, 8–9, 11; Fig. 1, *n* = 116) are presented in the supplementary file. No statistically significant difference was found between the two groups.

### Aim 2: Personal experiences of violence and asking questions about violence

Out of 592 participants, 121 (20.5%) reported having asked older adult patients about exposure to violence in the past 6 months, while 469 (79.5%) reported not having done so (item non-response *n* = 2, 0.3%). Responses were skewed, with only few respondents asking multiple times (Table 1). Multiple victimisation was associated with increased odds of asking questions about AOP (adj OR 2.45; 95% CI 1.14–5.28), while reports of either IPV or family violence were not. Among the background characteristics included in analysis, being a physician (adj OR 2.88 95% CI 1.46–5.68) or reporting “other” as occupation (adj OR 2.86 95% CI 1.42–5.74), as well as previous training on AOP (adj OR 1.82 95% CI 1.06–3.13) or domestic violence (adj OR 2.16 95% CI 1.36–3.41), were positively associated with asking older patients questions about AOP (Table 3).

### Aim 3: Negative feelings related to participation in training

Out of 439 respondents, 36 (8.3%) reported negative feelings to a very large extent on at least one item, and another 63 (14.5%) reported negative feelings to a large extent (items non-response *n* = 5, 1.1%). In total, 99 respondents (22.8%) reported negative feelings to a large or very large extent.

Experiences of IPV (OR = 2.98, 95% CI 1.54–5.77) and multiple victimisation (OR = 3.43 95% CI.1.43–8.21) were associated with reporting negative feelings related to participation in the training. Compared to assistant nurses, all other professions were less likely to report negative feelings: nurses OR = 0.31 (95% CI 0.18–0.53), physicians OR = 0.44 (95% CI 0.20–0.95), and other professions OR = 0.13 (95% CI 0.04–0.46).

As shown in Table 4, respondents who reported negative feelings also tended to perceive the training as valuable. For example, 27 respondents reported that the training as a whole had created negative feelings to a very large extent. However, 24 of those also perceived the training as valuable to a very large extent and two perceived the training as valuable to a large extent, while only one reported that the training was not valuable at all (Table 4).

**Table 3:** Multivariable logistic regression model for asking questions about violence (full sample, *n* = 592) and reporting negative effects (post-intervention sample, *n* = 439)

	Asking questions		Negative feelings	
	OR	95% CI	OR	95% CI
**Sex**				
Female	1		1	
Male	0.94	0.48–1.85	1.71	0.80–3.68
**Occupation**				
Assistant nurse	1		1	
Nurse	1.32	0.75–2.32	**0.31**	**0.18–0.53**
Physician	**2.88**	**1.46–5.68**	**0.44**	**0.20–0.95**
Other*	**2.86**	**1.42–5.74**	**0.13**	**0.04–0.46**
**Occupational experience**				
< 1 year	1		1	
1–5 years	1.27	0.54–3.02	1.22	0.45–3.29
5–10 years	1.10	0.44–2.71	0.96	0.34–2.71
> 10 years	1.21	0.53–2.76	1.09	0.42–2.82
**Previous training AOP**				
No, Don’t remember	1		1	
Yes	**1.82**	**1.06–3.13**	0.98	0.49–1.94
**Previous training DV**				
No, Don’t remember	1		1	
Yes	**2.16**	**1.36–3.41**	0.77	0.45–1.33
**Exposure to violence**				
No	1		1	
Family violence	1.70	0.93–3.13	1.21	0.58–2.53
IPV	0.99	0.51–1.94	**2.98**	**1.54–5.77**
Family violence and IPV	**2.45**	**1.14–5.28**	**3.43**	**1.43–8.21**

Notes: OR=Odds ratio; CI=Confidence Interval; AOP= Abuse of older people; DV= Domestic violence. IPV= Intimate partner violence. Bold numbers correspond to significant results

* Other included e.g. occupational therapists, physiotherapists, dietitians, counsellors and psychologist. Included in analysis asking questions *n* = 570 (missing cases = 22), Negative feelings = 422 (missing cases 17). Nagelkerke R^2^ model concerning asking questions = 0.11, concerning negative feelings = 0.16

**Table 4:** Crosstabulation between negative feelings and perceived value of the training

			Perceived training as valuable							
			Not at all		To a small extent		To a large extent		To a very large extent	
**Negative feelings**	n	%	n	%	n	%	n	%	n	%
**Training as a whole**										
Not at all	249	57.4	0	0	1	0.4	70	28.1	178	71.5
To a small extent	121	27.9	0	0	4	3.3	40	33.1	77	63.6
To a large extent	37	8.5	0	0	0	0	22	59.5	15	40.5
To a very large extent	27	6.2	1	3.7	0	0	2	7.4	24	88.9
**Forum theatre**										
Not at all	221	51.0	0	0	9	4.1	57	25.8	155	70.1
To a small extent	125	28.9	0	0	7	5.6	30	24.0	88	70.4
To a large extent	54	12.5	1	1.9	1	1.9	21	38.9	31	57.4
To a very large extent	33	7.6	0	0	0	0	6	18.2	27	81.8
**Videos and group discussions**										
Not at all	256	59.0	0	0	2	0.8	98	38.3	156	60.9
To a small extent	109	25.1	0	0	6	5.5	43	39.4	60	55.0
To a large extent	51	11.8	0	0	1	2.0	34	66.7	16	31.4
To a very large extent	18	4.1	0	0	0	0	1	5.6	17	94.4
**Lectures**										
Not at all	287	66.0	0	0	5	1.7	130	45.3	152	53.0
To a small extent	98	22.5	0	0	2	2.0	46	46.9	50	51.0
To a large extent	34	7.8	0	0	0	0	25	73.5	9	26.5
To a very large extent	16	3.7	0	0	0	0	1	6.3	15	93.8

Note: Only those responding to the item about negative feelings and the corresponding item about if the training was perceived as valuable were included in analysis. Missing values: Training as a whole *n* = 5 (1,1%), Forum theatre *n* = 6 (1,4%), Videos and group discussions *n* = 5 (1,1%) and Lectures *n* = 4 (0,9%)

## Discussion

This study investigated lifetime exposure to domestic violence among healthcare professionals, their patterns of asking patients about AOP, and reporting of negative feelings in relation to participation in AOP training. The results show that lifetime experiences of domestic violence are common among healthcare professionals, and that reporting multiple victimisation was associated with asking older patients questions about AOP. Experiences of IPV only or multiple victimisation were associated with a threefold increase in odds of reporting negative feelings in relation to participation in training. However, importantly, the vast majority of those reporting negative feelings in relation to the training also perceived the training as valuable.

The prevalence of victimisation should be compared between studies with caution, as the reported rates vary considerably due to methodological differences between the studies [15, 29]. We chose to consider violence by family perpetrators regardless of the age at victimisation; therefore, the reported prevalence of family violence is not directly comparable to the reported prevalence of childhood abuse in other studies. However, a previous study using a random population sample in Sweden found that the lifetime prevalence of physical, emotional, or sexual abuse by a parent, step-parent, or sibling was 11.4% for men and 14.9% for women [31]. Furthermore, a recent study of interpersonal violence in Arctic Sweden reported the lifetime prevalence of family violence among women to be 9.2–16.4% [34]. Hence our findings (men 14.7%, women 21.6%) indicate a higher prevalence of family violence in the current population of healthcare professionals. However, the difference found may well be attributed to a higher threshold for what was considered violent behaviour in the previous studies. For example, the previous studies used the phrase “systematically and over a long period of time” as a prerequisite for emotional violence, which we did not. The reported lifetime prevalence of IPV in the present study (men 8.8%, women 23.0%) is of the same magnitude as previous studies conducted both in the general population and among healthcare professionals in Sweden [29, 35, 37]. The prevalence in the total sample (21.2%) is almost exactly the same as the pooled prevalence reported among healthcare professionals in high-income countries in a global meta-analysis (20.7%) [10]. Previous studies have found that IPV may interfere with work performance [2, 10, 11] and that trauma exposure is associated with sickness absence [12, 17]. Although we did not investigate this association, the fact that almost one in four female respondents reported experiences of IPV emphasises the importance of employer awareness of IPV as a potential factor influencing both work life and the well-being of healthcare professionals. Therefore, IPV should be recognised as an occupational health and safety concern, and organisations needs to prioritise trauma-informed policies to support employees subjected to violence.

Previous studies have found varying associations between personal experiences of violence and the ability to identify and care for patients subjected to IPV [10]. In the two previous studies among Swedish healthcare professionals, personal experiences of IPV were not associated with asking patients direct questions about violence [35, 37]. However, these studies did not include personal experiences of family violence. In our study, we found that only those reporting both IPV and family violence were significantly more likely to ask questions about AOP (Table 3). Personal experiences of violence among healthcare professionals may increase awareness of victimisation as an underlying cause of ill health among patients, thereby increasing the likelihood of asking about abuse. This process may be particularly likely among professionals with more extensive abuse experiences. Multiple victimisation has repeatedly been associated with worse health outcomes and a higher risk of future victimisation than single victimisation [13, 16]. In this study, multiple victimisation was the only available indicator of abuse severity, as we did not measure frequency of exposure and included only one item per type of violence. This may explain why multiple victimisation, but not single forms of victimisation, was associated with asking questions about AOP. A more nuanced measure of severity might have yielded different result. It is also possible that there are varying associations between reporting personal experiences of different types of violence and asking questions about victimisation to different groups of patients. AOP differs compared to other forms of violence against adults, as it often involves both neglect and abuse by staff. When patients report these experiences, it may elicit different responses and emotions among healthcare professionals.

It has been estimated that AOP affects one in six community-dwelling older adults worldwide [44]. Considering that all respondents in the present study work closely with older patients, it is likely that they all have encountered patients who have been subjected to some form of abuse in the last six months. It is therefore concerning that only 20.5% reported that they have asked older patients about AOP. Still, these results align with our previous study, showing that only about half of healthcare professionals have *ever* asked an older patient about AOP [23], and with a random population sample, showing that only about 2% of respondents 65 years of age or older reported ever having been asked by healthcare professionals about experiences of abuse [30]. In this study, occupation was associated with asking patients about abuse, with assistant nurses less inclined to do so compared to physicians and those who fell into the “other” category (e.g., physiotherapists, occupational therapists, and counsellors). We reported a similar association in a previous study conducted in Sweden [23]. This finding is likely explained by differences in work tasks and education. Training on domestic violence is included in the educational curricula for most registered healthcare professions in Sweden, but not necessarily for assistant nurses. Furthermore, in the previous study, four out of five healthcare professionals attributed substantial responsibility to physicians for asking questions about abuse, whereas only one in three did so for assistant nurses [23]. This difference in perceived responsibility may well explain variations in asking about abuse between professions.

In this study, previous IPV or AOP training doubled the odds that healthcare professionals had asked questions about AOP, underlining the benefits of educating staff about AOP. Notably, only 8.3% of respondents reported negative feelings “to a very large extent” on at least one item concerning negative feelings, although the training involved elements that elicited emotions, including participatory theatre, videos, and case discussions. Importantly, almost all of those reporting negative feelings also perceived the training as valuable to a very large extent. This indicates that experiencing negative feelings while participating in a training about this sensitive topic does not necessarily mean that the training should be avoided. Instead, the reported negative feelings may reflect emotional engagement in the subject matter. Perceptions of the use of trigger warnings in higher education programmes have been previously investigated among medical students [6]. Opinions about whether trigger warnings should be used differed among the students, but all agreed that sensitive topics should not be avoided. This is also consistent with well-established principles that reminders of traumatic experiences should not be avoided simply because they may trigger negative reactions. Instead, avoidance of emotional or experiential reminders of trauma may lead to the prolongation of traumatic symptoms [40, 41]. Still, it is important to acknowledge that personal experiences of IPV or multiple victimisation were associated with a threefold increase in odds of reporting negative feelings. This aligns with previous findings that participating in training can lead to negative emotions, intrusive thoughts, and other post-traumatic symptoms for victims of violence [11, 28]. This emphasises the need to use a trauma-informed approach when training students or staff about addressing violence [5, 24]. Trauma-informed care is increasingly being implemented as the principal approach in the care of victims of violence ([3]; SAMHSA [27]). Central to such an approach is creating a safe environment for both patients and staff, reducing the risk of re-traumatisation and supporting the psychological safety of all parties. For educators, it is equally important to be aware that training may elicit memories of trauma among some participants and to design training in accordance with trauma-informed principles to ensure a safe environment for all [5, 28]. In this study, we used trigger warnings: participants were informed about the content of the training by e-mail beforehand, and the risk of the training triggering negative emotions was discussed as part of introduction on the day of the training. Participants were encouraged to be observant and supportive of their colleagues and were encouraged to contact the trainers (one of whom is a consultant psychiatrist with a completed foundation course in psychotherapy) if they wished to discuss emotions that were aroused during the training. This opportunity was utilised by a few participants. In addition, all participants were provided with contact information for societal resources for victims of violence and a phone number for the regional crisis therapist. It is possible that these steps contributed both to the low number of respondents reporting negative feelings and to the strong association found between reporting negative feelings and perceiving the training as valuable.

Assistant nurses were more likely to report negative feelings when participating in the training compared to other professionals, but they were also less likely to report asking questions about abuse. It is possible that these two results are related, as assistant nurses may have lower awareness of the issue and therefore be less likely to ask questions, while simultaneously being more affected by difficult content in the training. To gain a deeper understanding of the roots of negative feelings in relation to training about violence and to identify key aspects in designing trauma-informed training, additional research is needed, particularly studies using qualitative approaches.

### Strength and limitations

The present study has certain limitations that need to be considered. Firstly, the sample was not collected at random, which should be considered particularly when interpreting the reported prevalence rates. The total number of healthcare professionals employed at the participating clinics at the time of the intervention was not known, but management at the participating clinics aimed to schedule all employees to participate in the trainings, and we did not identify indications of systematic drop out. Hence, the number of professionals scheduled to participate is expected to be a good representation of the number of staff at the clinics. Also, sensitivity analyses showed no difference in reported prevalence between those participating together with their clinic or separately/only at baseline, also indicating that the results are representative. Potential participants were informed in advance about the training content, which may have led some to refrain from participating. If this was the case, both the prevalence rate and negative feelings during participation may have been underestimated. However, it is also possible that the information provided beforehand increased the willingness to participate among those with personal experiences of violence, potentially leading to an overestimation of prevalence rates. Previously, personal experiences of domestic violence have been found to be positively associated with attending domestic violence trainings [21]. Because the training was part of the continuing education programme, because the response rate was very high during the training day and because there was no statistical difference in reported prevalence in the sensitivity analyses, it is likely that the results are representative of healthcare professionals in Sweden. A substantial limitation is the lack of measure of abuse frequency or duration. Moreover, the number of perpetrators is unknown. “Multiple victimisation” was defined as reporting violence by both intimate partners and other family members; however, participants classified as having experienced violence by only one kind of perpetrator may still have been exposed to violence by several intimate partner or several family members. Another potential limitation is that only sex differences were accounted for in relation to prevalence estimates. It is likely that factors such as age, class, and ethnicity also affected the estimates, but investigating these aspects was beyond the scope of this study.

Data concerning respondents’ own exposure to violence was only included once. Hence, respondents completing those items at baseline may have been exposed to domestic violence between the baseline measurement and participating in the training, potentially influencing their responses regarding negative feelings and the perceived value of the training. However, as most respondents completed the items on own exposure during the morning of the training, this likely did not substantially affect the results.

Respondents were asked how many times they had asked older patients about abuse. Given the risk of recall bias over a six-month period, banded response categories would likely have been more appropriate. However, as responses were dichotomised for the analyses, alternative response categories would probably not have affected the results.

The study also has certain strengths that are important to consider. The design of the stepped wedge trial facilitated the ability to offer the educational intervention to many participants across several different workplaces. Consequently, the number of participants in this educational study was rather large. The stepped wedge design also reduced the risk of contextual bias when assessing the educational effects, as the intervention was provided in different environments and at different time points for various clusters of participants. For example, the influence of the COVID-19 pandemic on non-attendance was likely limited, as some clusters completed the educational intervention at the late phase of the COVID-19 pandemic (autumn 2021), while others did so at even later time points. Another strength was the detailed evaluation of the educational intervention. By including specific questions about different components of the training, the results yielded complex information about both the perceived value of the training and negative feelings. Such a detailed evaluation is useful, as experiences of participation in the training and previous victimisation can be complex.

## Conclusion

This study investigated the prevalence of exposure to violence among healthcare professionals and its association with asking older patients about abuse. Lifetime prevalence of domestic violence was 35.1% (IPV 21.2%, family violence 20.7%). Multiple victimisation was associated with asking older patients about AOP, indicating that personal experiences of violence among healthcare professionals may increase awareness and detection of AOP. Training in handling AOP was perceived as valuable, especially among staff who reported negative feelings during the training. This indicates that experiencing negative feelings during AOP trainings is not a contraindication for participation in training. Instead, these feelings may be a sign of emotional engagement due to the serious nature of the subject matter. A trauma-informed approach is essential when training healthcare professionals in handling AOP in order to prevent re-traumatisation among participants.

## Supplementary Information

Below is the link to the electronic supplementary material.

Supplementary Material 1

## Data Availability

The datasets used and analysed during the current study are available from the corresponding author on reasonable request.

## Abbreviations

AOP: Abuse of older people
CI: Confidence Interval
DV: Domestic Violence
IPV: Intimate partner violence
OR: Odds Ratio
REAGERA: Responding to Elder Abuse in GERiAtric care
