# Supplementary material for: Domestic violence among healthcare professionals in Sweden - Prevalence, associations with asking older patients about abuse and reactions to participating in training on abuse of older people
Source: BMC Health Serv Res. 2026 Aug 22;26:1177. doi: 10.1186/s12913-026-15440-y (PMC13508338; doi:10.1186/s12913-026-15440-y)
Supplement: Supplementary file 1 [file 12913_2026_15440_MOESM1_ESM.pdf]

Supplementary material

Prevalence of domestic violence for the total sample (n=592), for those participating in the training as part of a clinic (n=476) as well as for those only answering at baseline or participated in the training separately from their clinic (n=116).

	**Total sample**		**Participating with clinic**		**Only baseline or participating separately**		**p-value**
	**n**	**%**	**n**	**%**	**n**	**%**	
**Any domestic violence**							
Yes	203	35.1	162	34.8	41	36.3	0.76
No	376	64.9	304	65.2	72	63.7	
**Any IPV**	123	21.2	96	20.6	27	23.9	0.44
Physical IPV	50	8.6	40	8.6	10	8.8	0.93
Sexual IPV	37	6.5	30	6.5	7	6.4	0.96
Emotional IPV	96	16.7	76	16.5	20	17.9	0.72
Financial IPV	33	5.8	23	5.0	10	9.0	0.10
**Any family violence**	120	20.7	99	21.2	21	18.6	0.53
Physical family violence	75	13.0	65	13.9	10	8.8	0.15
Sexual family violence	26	4.6	19	4.1	7	6.4	0.31
Emotional family violence	59	10.3	51	11.0	8	7.1	0.22
Financial family violence	15	2.6	13	2.8	2	1.8	0.75*
**Multiple victimization**							
No violence	376	64.9	304	65.2	72	63.7	0.69
Only family violence	80	13.8	66	14.2	14	12.4	
Only IPV	83	14.3	63	13.5	20	17.7	
Both family violence and IPV	40	6.9	33	7.1	7	6.2	

Notes: IPV= Intimate partner violence. Item non-response for questions about exposure to violence: Physical violence n=13 (2.2%), Sexual violence n=21 (3.5%), Emotional violence n=18 (3.0%), Financial violence n=21 (3.5%).

P-values concerns differences in reported prevalence between respondents who participated as part of a clinic and for respondents who either only answered the baseline measure or participated in the training separately from their clinic.

** Pearson's chi square test was used to test for differences between the two groups of participants except for concerning financial family violence where Fischer's exact test was used.*
